# Intermediate alar resection: Precision technique for nasal tip aesthetics

**DOI:** 10.1016/j.jpra.2026.05.022

**Published:** 2026-05-17

**Authors:** Goran Latif Omer, Sureyya Şeneldir, Sahand Soran Ali, Giuseppe De Donato, Andrea Gravina, Sveva Viola, Gaia Grasso, Maria Grazia Maglie, Heshu Sulaiman Rahman, Othman Hussein, Aso Khasraw, Stefano Di Girolamo

**Affiliations:** a Department of Clinical Sciences, College of Medicine, University of Sulaimani, Sulaymaniyah, KRI, Iraq; b Department of Otorhinolaryngology, University of Rome Tor Vergata, 00133 Rome, Italy; c Süreyya Şeneldir Kliniği, İstanbul, Türkiye; d College of Pharmacy, American University of Iraq–Sulaimani, Sulaymaniyah, KRI, Iraq; e Royal Hospital, Sulaymaniyah, 46001, Iraq

**Keywords:** Rhinoplasty, Nasal tip surgery, Intermediate alar resection, Intermediate crus, Lower lateral cartilage, Tip refinement, Nasal tip projection, Nasal tip rotation, Boxy nasal tip, Patient-reported outcomes

## Abstract

**Objective:**

To evaluate the effectiveness of a newly described rhinoplasty technique, Intermediate Alar Resection (IAR), designed to refine the nasal tip by selectively modifying the intermediate crura.

**Methods:**

A retrospective cohort of 52 patients undergoing primary rhinoplasty with IAR between January 2024 and June 2025 was analysed. Standardised preoperative and postoperative photographs were assessed for nasolabial and nasofacial angles, Goode’s ratio, nasal projection, and tip width. Patient satisfaction was measured using the Rhinoplasty Outcome Evaluation (ROE) questionnaire, a validated six-item instrument scored from 0 to 24.

**Results:**

Postoperative analysis demonstrated significant improvements in nasal tip parameters. The median nasolabial angle increased from 97.1° to 105.6° (p < 0.001) and the nasofacial angle from 35.6° to 37.9° (p < 0.001). Tip width decreased by 12% on average, while nasal projection decreased by 7.5%. Goode’s ratio remained stable. Patient-reported outcomes indicated high satisfaction, with a median postoperative ROE score of 22 (IQR: 2) out of a maximum of 24, and no major complications were observed.

**Conclusion:**

Intermediate Alar Resection allows targeted modification of the intermediate crura, achieving predictable improvements in nasal tip projection, rotation, and definition. The technique is safe, effective, and associated with high patient satisfaction, offering a valuable addition to rhinoplasty methods.

## Introduction

Rhinoplasty is a surgical procedure aimed at improving both the form and function of the nose. Its outcomes depend on the underlying bony and cartilaginous framework, with the lower cartilaginous vault playing a central role in nasal tip support and aesthetics. This region is formed by the paired lower lateral cartilages (LLCs), whose configuration determines the shape of the tip and the appearance of the alar rims. The LLCs are classically divided into the medial crus (MC), intermediate crus (IC), and lateral crus (LC).^1^^,^^2^ Larrabee Jr. emphasised the importance of these structures in the tripod concept, which conceptualises the MCs as the central arm and the LCs as the lateral arms of a tripod. This model enhanced the understanding of nasal tip dynamics and guided the development of subsequent surgical techniques.^3^

Goldman first described the vertical dome division (VDD) in his landmark 1957 paper, introducing a technique that reshapes the nasal tip through a controlled vertical incision at the dome and repositioning of the mesial crura, enabling rotation, projection changes, and tip refinement without the use of implants or grafts.^4^ Simons later expanded on this work, coining the term ‘vertical dome division’ and advocating for mucosal preservation and more conservative cartilage excision, while also describing variations such as the “hockey stick” excision for overprojected tips.^5^ Lipsett similarly pioneered an approach to the lower cartilaginous vault involving transection at the level of the medial crura, with controlled shortening and reshaping via a chondroplastic flap technique that preserved the dome and lateral crus as a unit.^6^ Building further on these foundations, Şeneldir developed the vertical alar resection (VAR) technique. Unlike VDD, which largely preserved cartilage, VAR involves triangular resections of the LC to achieve more direct alterations in projection and rotation, producing stable improvements in nasal tip definition.^7^ In parallel, the columellar strut overlapping technique (COST) was proposed for cases with long or asymmetric MCs.^8^

Although these methods have been widely adopted, most focus on modifying either the MC or LC. The IC, despite its role in tip fullness and contour, has received relatively little dedicated attention in the literature.

Wise et al. described intermediate crural overlay (ICO), which targets the intermediate crus by dividing the LLC at the intermediate–medial crural junction and overlapping the intermediate crus over the medial crus with suture reapproximation, achieving deprojection while preserving the natural dome curvature and avoiding vertical dome division.^9^

The present study introduces the intermediate alar resection (IAR) technique, which specifically targets the IC for complete segmental cartilage excision in patients with long intermediate crura and a boxy-tip phenotype, followed by dome reconstruction and stabilisation within a structured support framework. Unlike ICO, IAR involves complete resection of the intermediate crus rather than an overlay manoeuvre, and is principally indicated for boxy-tip deformity associated with intermediate-segment hypertrophy. The method is adaptable: when necessary, resection may also include portions of the MC or LC, thereby serving as an alternative to COST or VAR in selected patients. By tailoring resection to the unique anatomy of each case, IAR offers precise control over projection, rotation, and definition, while providing a structural solution for wide domes and underrecognised IC-related deformities.

## Patients and methods

### Study design and setting

This study was a retrospective, single-arm cohort study, in which a newly described technique called Intermediate Alar Resection (IAR) was performed on 52 patients who attended the first author’s clinic for primary rhinoplasty from January 2024 to June 2025. Anamnesis, physical examination, and facial aesthetic measurements were completed for all patients. Photographs were taken preoperatively, intraoperatively, as well as one, two, six, and twelve months postoperatively. Facial lines and angles were measured from the photographs for each patient preoperatively and at the final follow-up visit, and the results were compared. Additionally, the Rhinoplasty Outcome Evaluation, a validated six-item patient-reported outcome questionnaire scored from 0 to 24, was completed by all patients one year after surgery. The ROE was administered in English.

### Participants

A total of 352 patients were assessed for eligibility. These patients presented with one or more of the following features: an over-projected nose, a broad (boxy) nasal tip, a wide nasal base, and/or an under-rotated (droopy) nose. Among these patients, many were subsequently excluded. The exclusion criteria included a history of rhinoplasty, a history of surgical or nonsurgical aesthetic facial interventions (before rhinoplasty or throughout the follow-up period), thin nasal skin as determined preoperatively by the Obagi “skin pinch test”,^10^ refusal to provide consent for the use of their photographs in the study, and those who did not complete the follow-up. In total, 52 patients met the inclusion criteria (Supplementary Figure 1). Only patients in whom intermediate alar resection was performed were included in the present analysis. All included patients completed the 12-month follow-up visit and postoperative photographic assessment.

Skin envelope thickness was systematically evaluated at the preoperative consultation using the Obagi “skin pinch test”, in which the skin overlying the nasolabial fold is gently pinched between the thumb and index finger to estimate dermal thickness, and patients are thereby classified as having thin, normal, or thick skin. Patients judged to have thin nasal skin were excluded a priori from the present cohort and managed with alternative techniques outside this study, on the rationale that complete intermediate-segment resection followed by dome reconstruction could, in a thin soft-tissue envelope, predispose to postoperative visibility of the dome reconstruction sutures or cartilage edges, supratip contour irregularities, and external nasal valve weakening secondary to insufficient soft-tissue camouflage and support. All 52 patients ultimately included in the present analysis were classified preoperatively as having normal or thick nasal skin.

### Surgical intervention

**Fig. 1:**
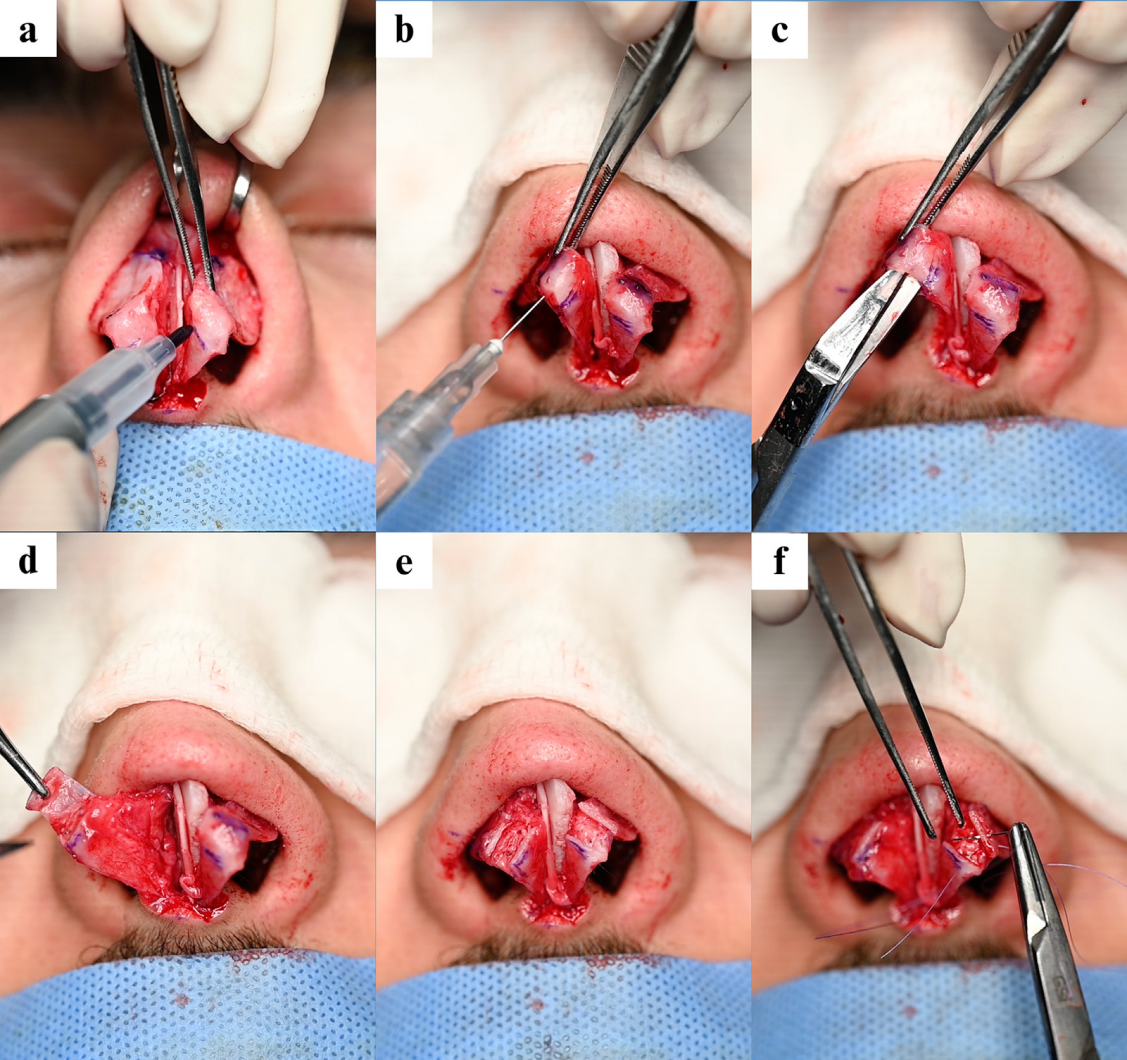
Intraoperative steps of Intermediate Alar Resection (IAR) in rhinoplasty. (a) The lower lateral cartilages are assessed and the crura junctions are marked. (b) The intermediate crus is injected with a lidocaine-epinephrine solution. (c) the intermediate crura are being dissected. (d) Intermediate crura are dissected, leaving the overlying mucosa and skin intact. (e) The lower lateral cartilages are exposed with the resected intermediate crura clearly visible. (f) The edges of the medial and lateral crura are reapproximated with 6-0 polydioxanone sutures to recreate the tip domes.

After confirming each patient’s fitness for anaesthesia, they were placed in the reverse Trendelenburg position and under general anaesthesia. The procedure began with infiltration of 5.4 mL of lidocaine-epinephrine (Persocaine-E) solution. An inverted V-shaped incision was then made at the narrowest point of the columella, followed by dissection of the lower lateral cartilages, nasal dorsum, and upper lateral cartilages. A hybrid de-humping was performed using a surgical drill, followed by septoplasty, graft harvesting, and medial and lateral osteotomies. Spreader grafts were then secured. The tip was assessed, and the LLCs were carefully reassessed intraoperatively. The lengths of the lateral, intermediate, and medial crura were noted individually and their junctions marked with a surgical pen (Fig. 1a). If a long intermediate crus was present (Fig. 1b), the intermediate crus was dissected (Fig. 1c) and excised through two incisions (Figs. 1d and 1e). In all variations of IAR, the resected segment was removed and the remaining medial and lateral crural edges were reapproximated using 6–0 polydioxanone sutures to recreate the nasal tip dome (Fig. 1f). Throughout IAR, the vestibular mucosa and overlying soft-tissue envelope were preserved.

### Intraoperative crural assessment and indication criteria for IAR

After exposure of the lower lateral cartilages, the MC, IC, and LC were reassessed intraoperatively and compared bilaterally. The MC–IC junction and the IC–LC junction were identified and marked to allow standardised segmental assessment. A long intermediate crus was defined as a disproportionate IC length relative to the ipsilateral MC and LC, in association with a boxy-tip phenotype. Because no universally accepted objective cut-off exists, this assessment was performed intraoperatively using a proportional surgeon-based classification.

### Variables

Photographs were obtained from seven standard views. Variables included: skin type; preoperative and postoperative nasolabial angle; nasofacial angle; Goode’s ratio; percentage change in tip width; and percentage change in nasal projection. All measurements were obtained using Fiji (ImageJ, version 1.54p, NIH), with linear variables normalised to stable facial reference distances.

### Data administration and statistical analysis

Statistical analyses were conducted using JASP (version 0.19.3). Continuous variables were tested for normality with the Shapiro–Wilk test. Age was normally distributed and summarised as mean ± SD; all other continuous variables are presented as medians with IQR. Differences between pre- and postoperative measurements were assessed using the Wilcoxon signed-rank test. Spearman’s rank-order correlation evaluated the relationship between age and tip-width change. Statistical significance was set at p < 0.05.

### Ethical considerations

All procedures were conducted in accordance with the Declaration of Helsinki. IRB approval was obtained from the Ethics Committee of the College of Medicine, University of Sulaimani (Approval Number 164). Written informed consent was obtained from all patients at the time of their preoperative consultation — prior to surgery — for participation in clinical research and for the use of their clinical data, measurements, and photographs for research and publication purposes. Consent was therefore prospective in nature, obtained at the outset of each patient’s clinical encounter as part of standard pre-operative documentation. This study is reported in accordance with the STROBE guidelines for observational studies.

## Results

A total of 52 patients were included in this study, with a mean age of 29.1 years (SD = 5.8). Female patients accounted for the majority of the cohort (78.8%, n = 41), while male patients comprised 21.2% (n = 11).

Significant postoperative improvements were observed in most measured angles. The nasolabial angle increased considerably after surgery, rising from a median preoperative value of 97.1° (IQR 13.6) to 105.6° (IQR 7.6) postoperatively (p < 0.001). In females, the nasolabial angle increased from 94.4° (SD 12.9) to 105.6° (SD 4.8), whereas in males it showed a slight decrease from 102.1° (SD 16.6) to 100.2° (SD 9.2). Similarly, the nasofacial angle showed a significant upward shift overall, with the median increasing from 35.6° (IQR 3.5) preoperatively to 37.9° (IQR 3.6) postoperatively (p < 0.001). In females, the nasofacial angle increased from 35.2° (SD 5.6) to 37.3° (SD 3.4), and in males from 37.1° (SD 2.6) to 40.2° (SD 2.4).

**Fig. 2:**
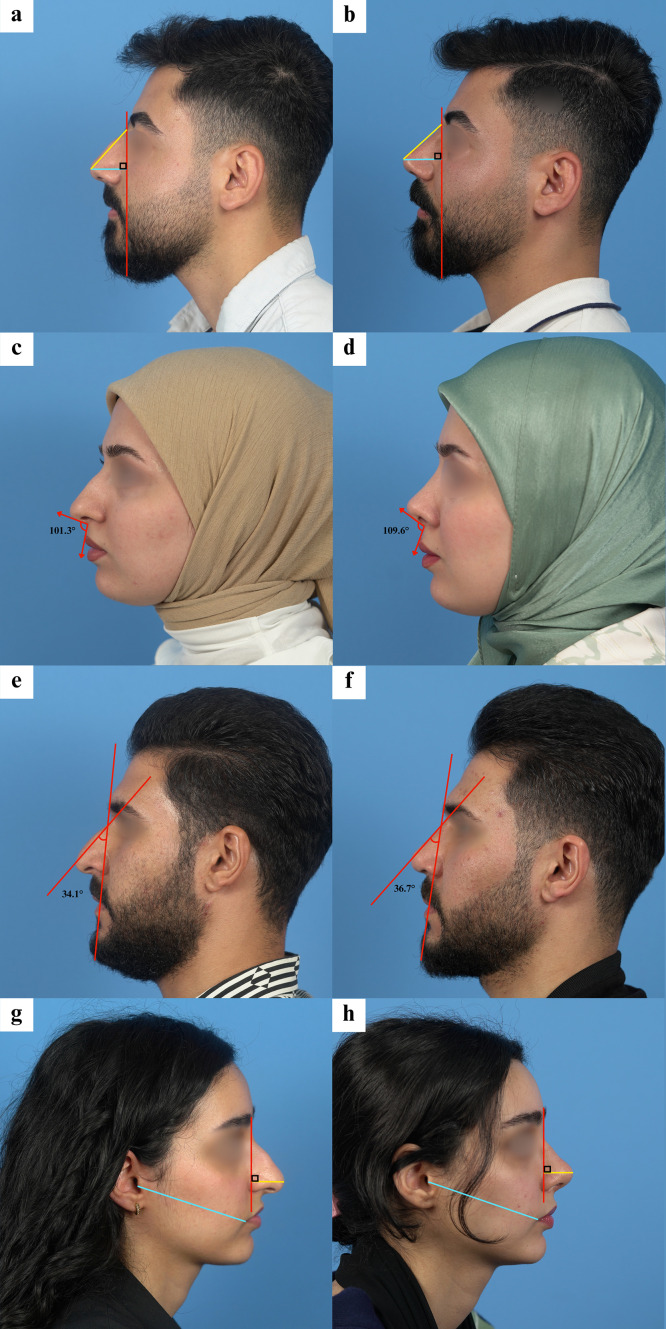

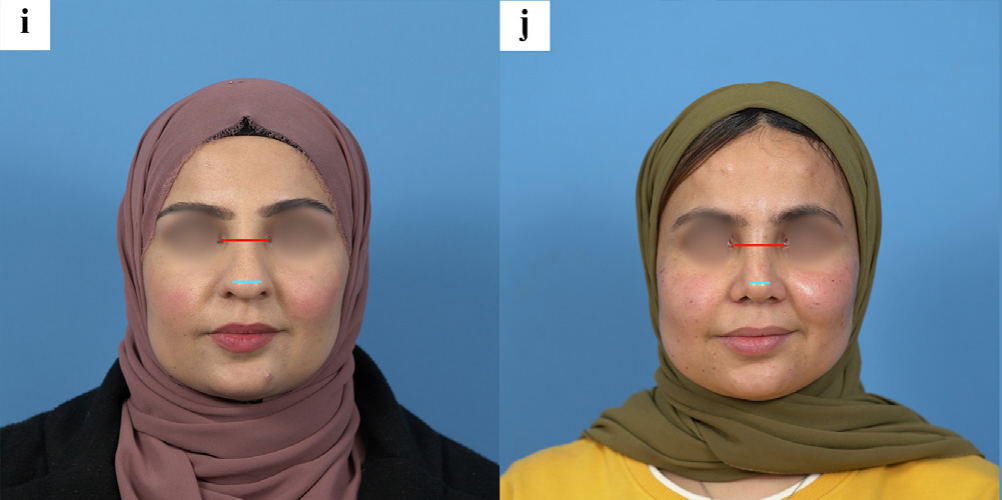
Preoperative and postoperative photographic views demonstrating key nasal tip measurements. (a, b) Left profile views illustrating Goode’s ratio measurement using nasal projection relative to nasal length. (c, d) Left profile views showing increase in nasolabial angle from 101.3° to 109.6° after intermediate alar resection. (e, f) Left profile views demonstrating nasofacial angle increase from 34.1° to 36.7°, reflecting improved nasal projection and facial harmony. (g, h) Right profile views of a patient showing the method for measuring nasal projection, standardized using the distance from the tragus to the oral commissure. (i, j) Frontal views illustrating the method of measuring nasal tip width relative to the intercanthal distance to ensure consistent scaling across photographs.

In contrast, Goode’s ratio remained stable. The median preoperative Goode’s ratio was 0.575 (IQR 0.08), and postoperative measurements showed a minimal change to 0.590 (IQR 0.02), which was not statistically significant (p = 0.640). Nasal projection decreased by approximately 7.5% after surgery, and tip width decreased by about 12% (Fig. 2). Age appeared to influence the extent of tip-width reduction, supported by a significant inverse correlation between age and percentage tip-width change (Spearman’s ρ = −0.297, p = 0.032).

No postoperative flattening or blunting of the infralobular break was observed. This was likely prevented by careful preservation of curvature during resection and precise dome reconstruction with 6–0 PDS sutures.

Patient satisfaction was assessed using the Rhinoplasty Outcome Evaluation (ROE) questionnaire, a validated six-item instrument scored from 0 to 24, with higher scores indicating greater satisfaction. The questionnaire was administered at the 12-month follow-up visit. The median postoperative ROE score was 22 (IQR: 2), indicating consistently high satisfaction across the cohort. Scores of 20 or above, considered indicative of excellent patient-reported outcomes, were achieved by the large majority of patients. No patient reported dissatisfaction with the functional or aesthetic result of surgery.

To illustrate the range of outcomes, Table 1 presents anonymised individual data from three representative cases: the most favourable, a near-mean representative, and the least favourable result in the cohort. The best outcome demonstrated a nasolabial angle increase of 28.6° and an 8.1% reduction in tip width. The representative case showed a 4.4° increase in nasolabial angle with a 16.0% reduction in tip width. Even in the least favourable case, positive changes in nasolabial angle (+2.6°) and tip width (−4.8%) were observed, consistent with the direction of group-level trends.

**Table 1:** Individual outcome data: best, representative, and least Favourable cases.

Case	Age	Sex	Pre NLA (°)	Post NLA (°)	ΔNLA (°)	Pre NFA (°)	Post NFA (°)	ΔNFA (°)	Pre GR	Post GR	ΔProj. (%)	ΔTip W. (%)
Best Outcome	21	F	83.0	111.6	+28.6	30.4	36.9	+6.5	0.53	0.58	12.6%	8.1%
Mean / Representative	30	F	97.4	101.8	+4.4	35.1	36.9	+1.8	0.55	0.55	0.6%	16.0%
Least Favourable	25	F	102.1	104.7	+2.6	39.9	37.7	−2.2	0.63	0.60	4.6%	4.8%

GR = Goode’s Ratio; NLA = nasolabial angle; NFA = nasofacial angle; Δ = postoperative change.

## Discussion

Optimal nasal tip refinement remains one of the most challenging aspects of rhinoplasty, requiring a balance between aesthetic goals and preservation of structural support. Over time, multiple techniques have been developed to adjust tip projection, rotation, and symmetry, reflecting evolving understandings of lower lateral cartilage anatomy. Vertical dome division (VDD), first described by Goldman in his 1957 landmark paper^4^ and evaluated in a long-term series by Davis et al.,^11^ emphasised conservative cartilage handling with repositioning of the medial crura to improve projection and rotation without implants. Simons refined and popularised VDD, coining the term and advocating for conservative cartilage excision.^5^ Vertical alar resection (VAR) produces more direct and stable changes in tip projection and rotation, with long-term stability of nasolabial and nasofacial angles reported by Şeneldir.^7^ Soylu’s versatile VAR (V-VAR) further supports individualised tip correction by varying the resection pattern according to the targeted crural segment.^12^ Modified VDD, as described by Gandomi et al., permits selective resection of medial, intermediate, or lateral crura with low revision rates, but is typically applied as part of a broader tip strategy rather than as a dedicated intermediate-crus-centred manoeuvre.^13^ Adamson et al. reported improvement in tip definition when addressing intermediate crus fullness, although their approach relied primarily on folding and suture-based modification rather than segmental excision.^14^

Prior efforts to address the intermediate crus specifically are also relevant. Lipsett described a chondroplastic flap technique involving medial crural transection and delivery, achieving controlled shortening and reshaping while preserving the dome and lateral crus as a unit.^6^ McCollough and English introduced the double-dome unit procedure as an alternative to the Goldman technique for the wide or bulbous lobule, using morselisation and suture reapproximation at the dome level rather than segmental resection.^15^ Soliemanzadeh and Kridel described an algorithm for nasal tip deprojection that includes medial crural overlay, whereby the medial crura are shortened by an overlay manoeuvre without excision.^16^ Wise et al. specifically described intermediate crural overlay (ICO), in which the LLC is divided at the intermediate–medial crural junction and the intermediate crus is overlapped over the medial crus with suture reapproximation, achieving deprojection without vertical dome division and preserving the natural dome curvature; ICO is particularly suited to thin-skinned patients with tip overprojection secondary to overdevelopment of the intermediate crura.⁹

Intermediate Alar Resection (IAR) shares with ICO the anatomical focus on the intermediate crus, but differs fundamentally in operative strategy. Whereas ICO achieves shortening by overlapping cartilage segments without excision and preserves the native dome, IAR involves complete segmental resection of the intermediate crus between its junctions with the medial and lateral crura, followed by dome reconstruction with 6–0 polydioxanone sutures and fixation to a tip-support construct. This difference in technique reflects a difference in indication: IAR is principally designed for patients with a boxy-tip phenotype associated with disproportionate intermediate-segment hypertrophy, in whom segmental excision and dome recreation offer more direct geometric correction than an overlay manoeuvre.

Other established approaches also contribute to contemporary tip management. Vertical alar folding (VAF) can address concave or over-projected lateral crura while preserving nasal valve function.^17^ Lateral crural steal has been associated with reliable increases in rotation and projection, although projection relapse may occur in some cases.^18^ COST combines lateral crural steal with medial crural overlap to simultaneously influence multiple tip parameters, supporting the concept of integrated, three-dimensional tip planning.^8^ Marianetti described monolateral crural overlay and dome truncation for deviated or asymmetric tips.^19^ Sazgar’s horizontal reduction with a cephalic hinged flap further highlights the value of conservative lateral crus techniques to narrow the tip and improve rotation while maintaining lateral support.^20^ Recent reviews similarly emphasise preserving cartilage integrity while tailoring correction to individual anatomy.^21^^,^^22^

In the present series, incorporation of IAR within a structured rhinoplasty framework was associated with significant improvements in nasal tip angles, reductions in tip width, and high patient-reported satisfaction at one year. The stability of Goode’s ratio despite reduced projection suggests proportional changes in nasal height related to dorsal reduction and/or tip rotation, thereby preserving overall nasal proportionality. The absence of major complications, including external nasal valve compromise or clinically evident tip instability, supports the feasibility of IAR when applied to appropriately selected patients.

Patient selection by skin type was a deliberate component of the present study and warrants particular emphasis. Using the Obagi pinch test,^10^ all included patients were classified preoperatively as having normal or thick nasal skin, and thin-skinned candidates were excluded a priori. This decision reflects well-recognised concerns that, in a thin soft-tissue envelope, complete intermediate-segment resection followed by dome reconstruction may translate into postoperative visibility of the dome reconstruction sutures or cartilage edges, supratip irregularities, and external nasal valve weakening due to inadequate soft-tissue support and camouflage. For thin-skinned patients with comparable IC-related deformities, alternative manoeuvres such as intermediate crural overlay, which preserves the native dome geometry without segmental excision,^9^ are likely more appropriate, and adjunctive soft-tissue camouflage with crushed cartilage or fascial grafts may be considered. The favourable safety profile of IAR observed in our cohort, in particular the absence of visibility-related complaints and external valve compromise, is therefore likely attributable not only to the surgical technique itself but also to the deliberate exclusion of unsuitable skin phenotypes at the consultation stage.

This study has limitations. The retrospective single-arm design and the presence of concomitant manoeuvres preclude direct attribution of all observed changes to IAR alone. In addition, no comparator cohort (for example, ICO, COST or VAR) was included, limiting conclusions regarding relative efficacy. Measurements were performed by a single non-blinded evaluator, which may introduce assessment bias. Multicentric comparative studies with standardised indication criteria and longer follow-up are needed to validate reproducibility, define durability, and assess generalisability across diverse patient populations.

## Conclusion

Intermediate Alar Resection (IAR) is a crural resection manoeuvre centred on the intermediate crus, an anatomical segment that has been relatively underemphasised in nasal tip surgery. Unlike intermediate crural overlay techniques that preserve the native dome through cartilage overlap, IAR involves complete segmental resection of the intermediate crus followed by dome reconstruction and fixation to a support construct, making it specifically suited to boxy-tip phenotypes associated with intermediate-segment hypertrophy. In this retrospective cohort, incorporation of IAR into primary rhinoplasty was associated with improvements in key nasal angles, consistent reductions in tip width, and high patient satisfaction at one year, without major complications. By allowing tailored resections of the intermediate, medial, and lateral crura when indicated, IAR may provide an additional option for surgeons managing selected nasal tip phenotypes. IAR is best applied to patients with normal or thick nasal skin, as determined preoperatively by the Obagi pinch test, and is not advised in thin-skinned patients, in whom the risk of contour visibility and external nasal valve compromise may be increased. Further comparative and multicentric studies are required to clarify its relative contribution compared with established techniques and to validate its applicability across diverse patient populations.

## Funding

This research received no specific grant from any funding agency in the public, commercial, or not-for-profit sectors.

## Patient consent

Written informed consent was obtained from all patients at the time of their preoperative consultation, prior to surgery, for participation in clinical research and for the use of their clinical data, measurements, and photographs for research and publication purposes.

## Ethical approval

The study was approved by the Ethics Committee of the College of Medicine, University of Sulaimani (Approval Number 164), and conducted in accordance with the Declaration of Helsinki.

## Declaration of competing interest

The authors declare that they have no competing interests.

## Appendix Supplementary materials

Image, application 1
