# Supplementary material for: Intermediate alar resection: Precision technique for nasal tip aesthetics
Source: JPRAS Open. 2026 May 17;50:365–74. doi: 10.1016/j.jpra.2026.05.022 (PMC13240758; doi:10.1016/j.jpra.2026.05.022)

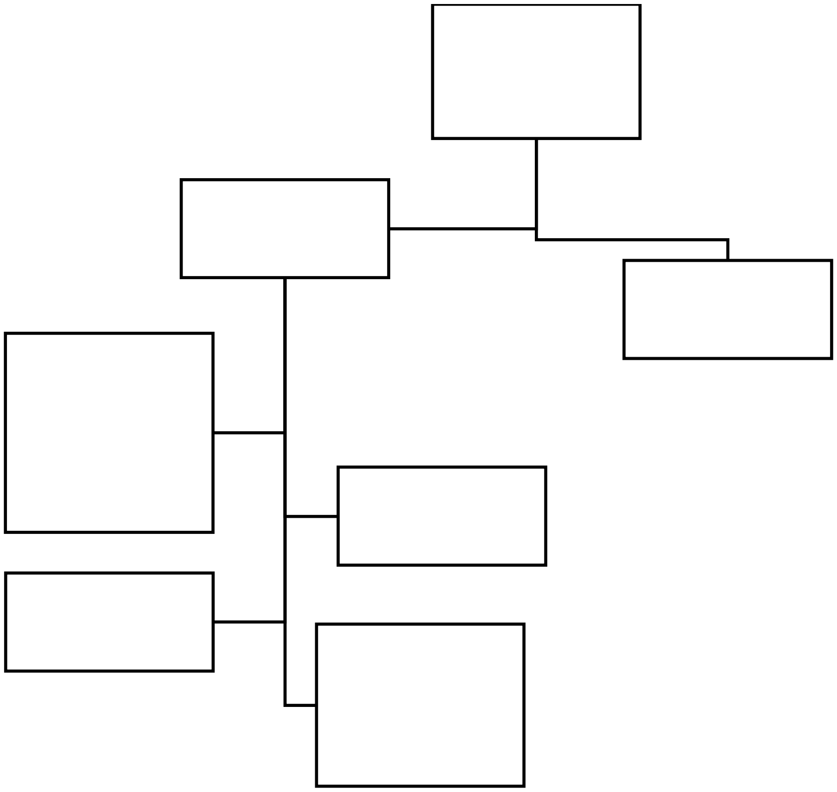

Supplement: Supplementary file 1 [file mmc1.docx]
